# Predictors of health-related quality of life in Parkinson’s disease: the impact of overlap between health-related quality of life and clinical measures

**DOI:** 10.1007/s11136-022-03187-y

**Published:** 2022-07-16

**Authors:** Itsasne Sanchez-Luengos, Olaia Lucas-Jiménez, Natalia Ojeda, Javier Peña, Juan Carlos Gómez-Esteban, María Ángeles Gómez-Beldarrain, Raquel Vázquez-Picón, Nerea Foncea-Beti, Naroa Ibarretxe-Bilbao

**Affiliations:** 1 Department of Psychology, Faculty of Health Sciences, University of Deusto, Bilbao, Spain; 2 Neurodegenerative Diseases Group, Biocruces Health Research Institute, Barakaldo, Spain; 3 Department of Neurology, Hospital of Galdakao, Galdakao-Usansolo, Spain

**Keywords:** HRQoL, Anxiety, Depression, Fatigue, Neurocognition, Motor symptoms

## Abstract

**Purpose:**

This study aimed to determine predictors of health-related quality of life (HRQoL) in Parkinson's disease (PD) and to explore their predictive value before and after controlling overlapping items between HRQoL and clinical variables.

**Methods:**

One hundred and eight PD patients underwent motor, anxiety, depression, apathy, fatigue, and neurocognition assessment. HRQoL was assessed by the Parkinson’s Disease Questionnaire-39 (PDQ-39). In order to determine predictors of HRQoL in PD, stepwise multiple regression analyses were performed in two ways: before and after removing the emotional well-being dimension from PDQ-39 to control the overlap between depression and anxiety, and HRQoL.

**Results:**

HRQoL total index was predicted by anxiety, fatigue, motor symptoms, and depression, explaining 26.9%, 7.2%, 2.8%, and 1.9% of the variance. However, after removing overlapping items, HRQoL total index was predicted by fatigue (16.5%), anxiety (6.1%), motor symptoms (3.9%), and neurocognition (2.5%), but not depression. Regarding HRQoL dimensions, mobility and activities of daily living were predicted by fatigue (19.7% and 5%) and UPDRS-III (4% and 10.2%); emotional well-being by fatigue (7.9%); social support by anxiety (12.2%) and UPDRS-III (8.6%); communication by neurocognition (5.3%) and UPDRS-III (3.4%); cognition by anxiety (10.6%) and bodily discomfort by anxiety (23%) and fatigue (4.1%).

**Conclusion:**

These findings showed the importance of identifying and controlling overlapping items of HRQoL and clinical measures to perform an accurate interpretation. HRQoL dimensions showed different predictors before and after controlling the overlap. Based on these results fatigue, anxiety, motor symptoms, and neurocognition, but not depression are the main predictors of HRQoL in PD patients.

**Supplementary Information:**

The online version contains supplementary material available at 10.1007/s11136-022-03187-y.

## Plain English summary

Parkinson’s disease is characterized by motor and non-motor symptoms that affect quality of life of Parkinson's disease patients. Health-Related Quality of Life of Parkinson's disease patients has been increasingly studied. Most studies are focused on the assessment of overall Health-Related Quality of Life by Parkinson’s Disease Questionnaire-39. Studies analyze the impact of motor and clinical symptoms, while cognition has not been thoroughly investigated. However, most studies in Parkinson's disease have not considered the possible overlap between clinical and quality of life measures. Additionally, few studies have investigated dimensions of the questionnaire. This study analyzed the influence of motor and non-motor symptoms on Health-Related Quality of Life in Parkinson's disease patients before and after controlling for the overlap between Health-Related Quality of life items and clinical variables. Our results suggest that overall Health-Related Quality of life was influenced by anxiety, fatigue, motor symptoms, and depression, being anxiety the predominant predictor. After controlling overlapping items, results showed that fatigue predominated over anxiety, motor symptoms, and depression. In fact, depression was no longer a predictor and neurocognition emerged as a predictor. Regarding dimensions, fatigue and motor symptoms, influenced mobility and activities of daily living dimensions. Fatigue influenced emotional well-being dimension. Anxiety and motor symptoms influenced social support dimension. Neurocognition and motor symptoms influenced communication dimension. Anxiety influenced cognition dimension, while bodily discomfort dimension was affected by anxiety and fatigue. These findings highlight the importance of identifying and controlling overlapping items of Health-Related Quality of life and clinical measures to perform an accurate interpretation of the results.

## Introduction

Assessment of patients’ quality of life has become an important outcome indicator in Parkinson’s disease (PD) [1]. PD patients show an impaired quality of life [2], not only due to the motor symptoms, but also several non-motor symptoms such as sensory abnormalities, sleep disorders, autonomic disturbances, cognitive impairment, and some neuropsychiatric aspects, like anxiety, depression, apathy, and fatigue [3, 4].

Quality of life includes both an individual's perception of themselves in the cultural context and the value systems, and the relationship to their goals, expectations, norms, and concerns [5]. Specifically, health-related quality of life (HRQoL) [2] is defined as the patient's life satisfaction, perception, and self-evaluation of the effects of a disease and their experience with it, being the physical and psychological dimensions, functional capacity, and social interactions the most relevant aspects [2, 6]. The way to assess HRQoL differs among studies and although there are several scales to measure this concept, Parkinson’s Disease Questionnaire-39 (PDQ-39) is the most commonly used instrument for HRQoL assessment in PD [2]. Most of the studies only analyze the overall HRQoL, while PDQ-39 was designed to assess different aspects of the functioning and well-being of people affected by PD [7]. This questionnaire is divided into eight dimensions: mobility, activities of daily living (ADL), emotional well-being, stigma, social support, cognition, communication, and bodily discomfort [7, 8]. Each of these dimensions offers the possibility of analyzing specific aspects that influence the HRQoL of PD patients [7, 8].

On the other hand, cognitive dysfunction is a non-motor symptom of PD that has an impact on the quality of life of PD patients [1]. Cognitive impairment is observed in 40% of people with PD during the course of the disease [9]. The most common deficits in PD involve visuospatial abilities, verbal memory, and frontal executive domains [10]. The risk of progression of cognitive impairment to dementia has a functional impact on the ADL and HRQoL of people with PD [6, 11, 12]. The high association between cognitive impairment and difficulties in ADL persists with disease progression [12] and 83% of PD patients with cognitive impairment develop dementia after 10–20 years [11].

Anxiety, depression, apathy, and fatigue are other characteristic non-motor symptoms that can affect the quality of life of PD patients [13–16]. Neuropsychiatric problems are present in more than 77% of people with PD [17]. Specifically, meta-analyses showed that the average prevalence rate of anxiety disorders in PD patients was 31% [18], depressive symptoms were found in 22.9% [19], apathy in 40% [16], and fatigue in 50% [14]. Although research generally focuses on depressive symptoms, anxiety is a very common symptom in PD [18] and both have been reported to affect the quality of life of people with PD [15, 20, 21].

A systematic review showed that HRQoL of PD patients has been increasingly explored through diverse studies investigating the predictors of the overall HRQoL in PD [22]. It is essential to identify the most important predictors of HRQoL in PD patients in order to set treatment priorities and reduce the effects of the functional and emotional consequences of PD [22]. However, it should be considered that measures of depression and quality of life are concepts with overlapping items [23–26], because emotional distress is a construct that is present in most quality of life questionnaires [23, 24], including specific items assessing psychopathology [24, 25], such as depressive [23, 25] and anxiety symptoms. As suggested by Hays and Fayers [23], most of the articles published to date in different pathologies have included measures of depression and HRQoL without considering the empirical and conceptual overlap between the two variables [23]. Therefore, not taking into account the overlap between these measures may result in tautological inferences about the influence of depression on HRQoL, since, if a large proportion of the items overlap, it is logical that quality of life would be significantly associated with measures of psychopathology [23, 24]. Recognition of the overlap between these variables is crucial to interpret associations and avoid incorrect causal inference [23], so it is recommended to control for the presence of psychopathological items in quality of life measures [24].

Conversely, predictors of HRQoL dimensions have not been studied as much as predictors of overall HRQoL [13, 17, 27–31]. Moreover, cognition has not been studied as often as the clinical and motor variables of the disease. Despite some studies having analyzed cognition as a predictor of overall HRQoL [6, 21, 28–30, 32–35], few of them have examined cognition as a predictor of the specific dimensions of HRQoL [28–30]. Most of these studies have analyzed cognition with cognitive functioning screening tests, however, further research is needed to analyze the impact of cognition on overall HRQoL and its dimensions using a comprehensive neuropsychological assessment.

Therefore, the main goal of this study was to simultaneously analyze the predictive value of a wide spectrum of variables such as anxiety, depression, apathy, fatigue, cognition, and motor symptoms that impact HRQoL and its dimensions. The second aim of this study was to observe the predictive value of these variables after identifying and controlling the overlap between HRQoL items and clinical measures.

## Methods

### Participants

One hundred and fifteen PD patients participated in the study between December 2016 and February 2020. Participants were recruited via: (1) the neurologists from the Hospital of Galdakao and the Cruces University Hospital involved in the study, who recruited outpatients regularly attending appointments at the hospital; and (2) ASPARBI and ASOPARA Parkinson's Associations, where informative talks were organized and patients who were members of the associations were recruited. Written informed consent was obtained from all subjects after receiving an explanation of the study. This study was conducted according to the guidelines of Strengthening the Reporting of Observational studies in Epidemiology (STROBE) [36].

The diagnosis of PD based on the diagnostic criteria of the UK PD Society Brain Bank was made by a movement disorder specialist [37]. Other inclusion criteria were as follows: (1) age ≥ 45 and (2) either male or female. The exclusion criteria were as follows: (1) diagnosis of dementia as defined by DSM-IV-TR [38] and the Movement Disorders Society (MDS) specific clinical criteria for PD dementia [39], and (2) diagnosis of other major neurological or psychiatric disorders. All patients received their pharmacological treatment and all evaluations were done in the on medication state. Of the 115 participants, four participants were excluded as a result of other diagnoses (three for atypical Parkinsonism disorders and one for Alzheimer's disease) and three participants dropped out of the study. Finally, the sample was composed of 108 participants.

### Demographic, cognitive reserve, and PD-related features assessment

Demographic information, such as age, sex, years of education, and Cognitive Reserve Questionnaire [40] were included. Levodopa Equivalent Daily Dose (LEDD) [41] and years of disease duration were registered. Estimation of the stage and course of the disease was assessed by the Hoehn and Yahr stage Scale (H&Y) [42]. According to MDS level I criteria [43], mild cognitive impairment (MCI) was classified using Montreal cognitive assessment (MoCA) scores, adjusted for age and years of education based on the normative data for the Spanish population [44]. The prevalence in the sample was 56.5%.

### Outcome measure

HRQoL was assessed with the Spanish version of PDQ-39 [8]. This questionnaire is divided in eight dimensions: mobility (10 items, 0–40), ADL (6 items, 0–24), emotional well-being (6 items, 0–24), stigma (4 items, 0–16), social support (3 items, 0–12), cognition (4 items, 0–16), communication (3 items, 0–12), and bodily discomfort (3 items, 0–12). The scores for each dimension were calculated by the sum of the raw scores of each item divided by the maximum possible score of each dimension and multiplied by 100 [45]. The PDQ-39 summary index (HRQoL total index) was calculated by the sum of the scores of the 8 dimensions and divided by the number of dimensions [45]. Scores ranged from 0 (no problem) to 100 (maximum level of problems), showing that the higher the score, the lower the HRQoL [45]. An overlap between the items of depression and anxiety measures of the Hospital Anxiety and Depression Scale (HADS) and the emotional well-being dimension items of the PDQ-39 was found (Table 1), so a new HRQoL total index was created without the overlapping items of the emotional well-being dimension.

**Table 1:** Items excluded from PDQ-39 due to the similarity with the HADS scale

PDQ-39 questionnaire: emotional well-being dimension	
17. Felt depressed 18. Felt isolated and lonely 19. Felt weepy or tearful 20. Felt angry or bitter 21. Felt anxious 22. Felt worried about the future	

*PDQ-39* Parkinson’s Disease Questionnaire-39

### Potential determining variables

#### Motor symptoms assessment

Motor symptoms were assessed by the Unified Parkinson's Disease Rating Scale (UPDRS-III) [46].

#### Anxiety, depression, apathy, and fatigue assessment

Anxiety and depression were measured with the Spanish version of HADS [47], divided into two subscales: seven items of anxiety and seven items of depression. Apathy scores were evaluated with the Spanish version of Lille Apathy Rating Scale (LARS) [48] composed of 33 items. Physical and mental fatigue scores were assessed using the 9-item self-administered Fatigue Severity Scale (FSS) [49].

#### Neuropsychological assessment

Participants performed an extensive neuropsychological battery in which different cognitive functions were evaluated. Due to the use of multiple measures to assess different cognitive domains, a neurocognition composite score was created including attention and working memory (Digits forward and backward subtest from the Wechsler Adult Intelligence Scale-III [50]), verbal memory (Hopkins Verbal Learning Test-Revised, long-term recall and learning [51]), visual memory (Brief Visual Memory Test-Revised, long-term recall and learning [52]), semantic (Calibrated Ideational Fluency Assessment-Animals category [53]) and phonemic fluency (MoCA language category-fluency subtest [54]), executive functions (UD interference test, Words-Colors and interference [55]), visuoconstructive abilities (Clock Drawing Test, order and copy [56]), cognitive flexibility (Modified Wisconsin Card Sorting Test, categories, and perseverative errors [57]), and processing speed (Salthouse Letter Comparison Test [58] and Trail Making Test-A [59]). All cognitive measures were converted into *z* scores, and the sign of some measures was adjusted so that higher scores indicated higher cognitive performance. The neurocognition composite score showed a satisfactory internal consistency (*α* = 0.87).

### Statistical analysis

The Kolmogorov–Smirnov test was performed to test normality of data. Missing values were imputed using multiple imputation with the Expectation–Maximization (EM) algorithm. The percentage of missing values was 0.047%.

Spearman’s rho test correlation was used. Firstly, the correlation of HRQoL total index (before and after overlapping items) and its dimensions, with demographic (age and sex), cognitive reserve and LEDD was assessed. These variables were used to adjust the regression models in step 1 as covariates only in the cases that significantly correlated with the HRQoL total index (before and after overlapping items) and the different HRQoL dimensions. The H&Y scale and years of disease duration in PD patients were not included in the model as they were considered global measures of PD severity [60]. Secondly, the relationship of HRQoL total index (before and after overlapping items) and its dimensions, with the potential determining variables (UPDRS-III, anxiety, depression, apathy, fatigue, and neurocognition composite) was assessed. Due to the overlap of items between the emotional well-being dimension and HADS scale, the correlation between the anxiety and depression scores with the emotional well-being dimension was not considered.

Stepwise Multiple Regression analyses were conducted including those variables that correlated significantly in the previous correlation test with the HRQoL total index (before and after overlapping items) and HRQoL dimensions. Regression analyses were adjusted in step 1 (enter method) by covariates showing significant correlations. In step 2 (forward method) motor symptoms, anxiety, depression, apathy, fatigue, and neurocognition scores were included in the models. The statistical analyses were carried out using IBM SPSS statistics v27.

## Results

Demographic, cognitive reserve, PD-related features, HRQoL, anxiety, depression, apathy, fatigue, and neurocognition scores of the sample are provided in Table 2.

**Table 2:** Demographic characteristics, cognitive reserve, PD-related features, HRQoL, anxiety, depression, apathy, fatigue, and neurocognition composite score of the study sample (*n* = 108)

	Mean/*n*	SD/%	95% CI
Age (years)	70.15	7.94	68.63 to 71.66
Sex (male)	75	69.4%	
Years of education	10.81	4.45	9.97 to 11.66
Cognitive reserve	11.15	3.97	10.39 to 11.91
*Hoehn & Yahr*			
Stage 1.0	20	18.5%	
Stage 1.5	14	13%	
Stage 2.0	41	38%	
Stage 2.5	6	5.6%	
Stage 3.0	25	23.1%	
Stage 4.0	2	1.9%	
Years of disease duration	6.35	4.94	5.41 to 7.29
LEDD	600.73	412.63	522.02 to 679.44
UDPRS III	20.61	11.80	18.36 to 22.86
*HRQoL total index (PDQ-39 summary index)*^*a*^	20.75	10.88	18.68 to 22.83
Mobility^a^	23.24	20.56	19.32 to 27.16
ADL^a^	21.26	18.51	17.73 to 24.79
Emotional well-being^a^	28.55	23.18	24.13 to 32.97
Stigma^a^	9.66	14.93	6.81 to 12.51
Social support^a^	8.02	14.98	5.17 to 10.88
Cognition^a^	25.75	16.98	22.51 to 28.99
Communication^a^	13.66	15.85	10.63 to 16.68
Bodily discomfort^a^	35.88	25.84	30.95 to 40.80
*HADS-A*	6.16	4.46	5.31 to 7.01
Scores > 8	40	37%	
*HADS-D*	4.84	3.65	4.15 to 5.54
Scores > 8	24	22.2%	
*LARS*	− 23.38	6.55	− 24.63 to − 22.13
Scores > − 14	9	8.3%	
*FSS*	30.57	15.72	27.58 to 33.57
Scores > 36	43	39.8%	
Neurocognition composite score^b^	− 0.00	0.67	− 0.13 to 0.13

*SD* standard deviation, *CI* confidence interval, *H&Y* Hoehn and Yahr stage Scale, *LEDD* Levodopa Equivalent Daily Dose, *UPDRS III* The Unified Parkinson's Disease Rating Scale-motor part, *HRQoL* Health-Related Quality of Life, *ADL* Activities of Daily Living, *HADS-A* Hospital Anxiety and Depression Scale-Anxiety scores, *HADS-D* Hospital Anxiety and Depression Scale-Depression scores, *LARS* Lille Apathy Rating Scale, *FSS* Fatigue Severity Scale

^a^Scores from 0 to 100

^b^Average in *z*-scores

### Correlation analyses of HRQoL and its dimensions

Cognitive reserve was correlated with HRQoL total index (before and after overlapping items), mobility, and emotional well-being dimensions; LEDD variable with HRQoL total index after removing overlapping items, mobility, and communication dimensions; age with mobility dimension and sex with HRQoL total index, emotional well-being, and bodily discomfort dimensions. The correlation coefficients showed weak associations. Consequently, cognitive reserve, LEDD, age and sex variables were included as covariates in the models whose correlation was significant (Table 3).

**Table 3:** Spearman's rho correlation analyses between HRQoL and demographic characteristics, cognitive reserve and LEDD of the study sample

Criterion variable	HRQoL total index	HRQoL without overlap	Mobility	ADL	Emotional well-being	Stigma	Social support	Cognition	Communication	Bodily discomfort
Age	0.14	0.12	0.25*	0.05	0.09	− 0.09	− 0.04	0.15	0.11	0.03
Sex	0.21*	0.15	0.14	0.00	0.27*	0.10	0.06	0.12	− 0.10	0.24*
Cognitive reserve	− 0.29*	− 0.26*	− 0.29*	− 0.16	− 0.22*	− 0.02	− 0.11	− 0.08	− 0.15	− 0.13
LEDD	0.15	0.19*	0.22*	0.05	0.03	0.08	0.07	− 0.03	0.28*	0.13

*HRQoL* health-related quality of life, *ADL* activities of daily living, *LEDD* Levodopa Equivalent Daily Dose

∗*p* ≤ 0.05

Regarding the potential determining variables, in general, weak to moderate significant correlations were found between HRQoL total index (before and after overlapping items), several dimensions of HRQoL, and motor symptoms, anxiety, depression, fatigue, and neurocognition scores (Table 4 and Supplementary Material S1).

**Table 4:** Spearman's rho correlation analyses between HRQoL and potential determining variables: motor symptoms, anxiety, depression, apathy, fatigue, and cognition of the study sample

Criterion variable	HRQoL total index	HRQoL without overlap	Mobility	ADL	Emotional well-being	Stigma	Social support	Cognition	Communication	Bodily discomfort
*Motor symptoms*										
UPDRS-III	0.23*	0.25*	0.28*	0.30*	0.06	0.09	− 0.25*	0.17	0.29*	0.19*
*Anxiety, depression, apathy and fatigue scores*										
HADS-A	0.52*	42*	0.31*	0.03	0.70*	0.06	0.22*	0.33*	0.18	0.48*
HADS-D	0.50*	0.43*	0.42*	0.19*	0.49*	0.09	0.27*	0.24*	0.08	0.33*
LARS	0.18	0.17	0.13	0.16	0.12	0.02	0.01	0.18	0.10	0.02
FSS	0.43*	0.44*	0.44*	0.22*	0.26*	0.10	0.02	0.13	0.25*	0.38*
*Cognition*										
Neuro-cognition^a^	− 0.26*	− 0.24*	− 0.31*	− 0.15	− 0.20*	− 0.11	− 0.01	− 0.19*	− 0.19*	− 0.10

*HRQoL* Health-Related Quality of Life, *ADL* Activities of Daily Living, *UPDRS III* The Unified Parkinson's Disease Rating Scale-motor part, *HADS-A* Hospital Anxiety and Depression Scale-Anxiety scores, *HADS-D* Hospital Anxiety and Depression Scale-Depression scores, *LARS* Lille Apathy Rating Scale, *FSS* Fatigue Severity Scale

^a^Average in *z*-scores

^*∗*^*p* ≤ 0.05

### Predictors of HRQoL and its dimensions

Tolerance values (> 0.1) and variance inflation factor (VIF) values (< 10) of all predictor variables were appropriate, showing that there was no issue with the multicollinearity data.

Stepwise Multiple Regression analyses indicated that the overall model of HRQoL was significant (*F* (6,101) = 17.77; *p* < 0.001), showing that HRQoL total index was predicted by scores of anxiety (*β* = 0.32; *p* = 0.001), fatigue (*β* = 0.24; *p* = 0.003), UPDRS-III (*β* = 0.17; *p* = 0.021), and depression (*β* = 0.18; *p* = 0.050). When emotional well-being dimension overlapping items were removed, fatigue (*β* = 0.33; *p* < 0.001), anxiety (*β* = 0.25; *p* = 0.005), UPDRS-III (*β* = 0.20; *p* = 0.015), and neurocognition (*β* = − 0.20; *p* = 0.038) were predictors of HRQoL total index, and depression was no longer significant (Table 5).

**Table 5:** Stepwise multiple regression analyses of HRQoL total index, HRQoL dimensions, anxiety, depression, apathy and fatigue scores, neurocognition composite score and motor symptoms of the study sample

Criterion variable	*F*	*R*^2^ (%)	*R*^2^ change (%)	*β*	*t*	*p*	Predictors
*HRQoL total index before and after removing emotional well-being dimension*							
Model 1^a^	17.77	51.3					
HRQoL total index			26.9	0.32	3.46	0.001	HADS-A
			7.2	0.24	3.09	0.003	FSS
			2.8	0.17	2.35	0.021	UPDRS-III
			1.9	0.18	1.99	0.050	HADS-D
Model 2^b^	12.42	42.5					
HRQoL total index without emotional well-being dimension			16.5	0.33	3.84	0.000	FSS
			6.1	0.25	2.86	0.005	HADS-A
			3.9	0.20	2.46	0.015	UPDRS III
			2.5	− 0.20	− 2.10	0.038	Neurocognition
*HRQoL dimensions*							
Model 3^c^	11.83	36.7					
Mobility			19.7	0.41	5.09	0.000	FSS
			4.0	0.21	2.54	0.013	UPDRS III
Model 4	9.37	15.1					
ADL			10.2	0.27	2.95	0.004	UPDRS III
			5.0	0.23	2.48	0.015	FSS
Model 5^d^	9.71	21.9					
Emotional well-being			7.9	0.28	3.24	0.002	FSS
Model 6	13.80	20.8					
Social support			12.2	0.39	4.44	0.000	HADS-A
			8.6	− 0.30	− 3.38	0.001	UPDRS III
Model 7	12.51	10.6					
Cognition			10.6	0.33	3.54	0.001	HADS-A
Model 8^e^	7.26	17.3					
Communication			5.3	− 0.23	-2.54	0.013	Neurocognition
			3.4	0.19	2.08	0.040	UPDRS III
Model 9^f^	17.76	33.9					
Bodily discomfort			23.0	0.41	4.74	0.000	HADS-A
			4.1	0.22	2.55	0.012	FSS

*HRQoL* Health-Related Quality of Life, *ADL* Activities of Daily Living, *UPDRS III* The Unified Parkinson's Disease Rating Scale-motor part, *HADS-A* Hospital Anxiety and Depression Scale-Anxiety scores, *HADS-D* Hospital Anxiety and Depression Scale-Depression scores, *FSS* Fatigue Severity Scale

^a^Adjusted by cognitive reserve and sex [Significant in model 1 in cognitive reserve (*β* = − 0.16; *p* = 0.047)]; ^b^Adjusted by cognitive reserve and Levodopa Equivalent Daily Dose (LEDD)

^c^Adjusted by cognitive reserve, age and LEDD [Significant in model 3 in cognitive reserve (*β* = − 0.26; *p* = 0.003)]

^d^Adjusted by cognitive reserve [Significant in model 5 (*β* = − 0.21; *p* = 0.024)] and sex [Significant in model 5 (*β* = 0.24; *p* = 0.013)]

^e^Adjusted by LEDD [Significant in model 7 (*β* = 0.26; *p* = 0.006)]

^f^Adjusted by sex [Significant in model 8 (*β* = 0.18; *p* = 0.032)]

Regarding regressions of HRQoL dimensions, mobility dimension was predicted by fatigue scores (*β* = 0.41; *p* < 0.001) and UPDRS-III (*β* = 0.21; *p* = 0.013). ADL dimension was predicted by UPDRS-III (*β* = 0.27; *p* = 0.004) and fatigue scores (*β* = 0.23; *p* = 0.015). Emotional well-being dimension was predicted by fatigue scores (*β* = 0.28; *p* = 0.002). Social support dimension was predicted by anxiety scores (*β* = 0.39; *p* < 0.001) and UPDRS-III (*β* = − 0.30; *p* = 0.001). Cognition dimension was predicted by anxiety scores (*β* = 0.33; *p* = 0.001). Communication dimension was predicted by neurocognition (*β* = − 0.23; *p* = 0.013) and UPDRS-III (*β* = 0.19; *p* = 0.040). Bodily discomfort dimension was predicted by anxiety (*β* = 0.41; *p* < 0.001) and fatigue scores (*β* = 0.22; *p* = 0.012) (Table 5). Stigma dimension did not show significant correlations, so their regression analyses were not performed (Table 4).

## Discussion

The aim of this study was to investigate the predictors of HRQoL and its dimensions in PD patients. Specifically, this study is a multidimensional predictive model that analyzes the predictor value of motor and non-motor symptoms, including scores of anxiety, depression and fatigue and neurocognitive functions in HRQoL and its dimensions in PD. Moreover, this study analyzed the predictive value of these variables after considering the overlapping items between HRQoL and clinical measures.

This study has identified several characteristics associated with a worse HRQoL in PD patients. Higher scores for anxiety, fatigue, motor symptoms, and depression were the factors that predicted a worse HRQoL, with anxiety being the main predictor. The variance in the predictive value of depression scores was lower than in the other studies, with anxiety, fatigue, and motor symptoms predominating over depression scores. Most studies showed that depression was the main predictor of HRQoL [22], and it may be due to the absence of anxiety and depression variables joined in the same regression model [21]. Additionally, the high association between depressive symptoms and quality of life may be due to the overlap between the emotional items of the quality of life measures and depressive symptomatology [25], so these results should be interpreted with caution. Therefore, in our study, an additional analysis was performed in which the emotional well-being dimension items of the HRQoL total index were removed to consider this overlap. Interestingly, our results showed that after taking into account the overlap, fatigue predominated over anxiety and motor symptoms; but depression was no longer a predictor of HRQoL total index. Most studies have not considered the overlap between clinical and HRQoL measures in their analyses and it may be the main reason why depression has been considered as the main predictor of HRQoL. Future studies should contemplate the overlap between clinical and HRQoL measures to perform more accurate interpretations. Controlling the overlapping, fatigue was the main predictor of HRQoL total index. Fatigue is recognized as one of the most disabling symptoms of PD [61], present in 50% of PD patients [14], however, it has not been such a studied predictor as anxiety and depression. In four of the five studies reviewed in a systematic review, fatigue was a strong predictor of HRQoL [22], demonstrating that it emerges in the early stages of PD and persists throughout the course of the disease, negatively impacting their HRQoL [14]. Additionally, in our study, neurocognition emerged as a predictor of HRQoL total index when controlling the overlap. The majority of the studies analyzed cognition with cognitive functioning screening tests [21, 28, 29, 32–35]. In contrast, in our study, participants performed an extensive neuropsychological battery comprised of a wide variety of cognitive tests, revealing that neurocognition composite score predicted HRQoL. To the authors´ knowledge, only four studies found cognition as a predictor of HRQoL total index [6, 29, 30, 33]. Two of these studies analyzed cognition with cognitive functioning screening tests [29, 33], while the two other studies assessed cognition by specific cognitive measures, showing that working memory [30], verbal fluency [30], visual attention/memory [6], visuospatial [6], and executive functioning [6] were predictors of HRQoL. As far as the authors are aware, this is the first study investigating predictors of HRQoL in PD patients in which the possible overlap between HRQoL and clinical outcomes was analyzed. Furthermore, it should be emphasized that the methodology used and the results obtained in this study could be extrapolated to other pathologies.

Regarding the specific HRQoL dimensions, mobility and ADL dimensions were predicted by fatigue and UPDRS-III. Similar to our results, other studies found that reduced activity and motor symptoms were associated with mobility [27] and ADL dimension [27, 28, 31], while physical fatigue was also a predictor of mobility dimension [62]. Consistent with previous studies [27], our findings showed that fatigue was also a predictor of emotional well-being dimension. Other research suggest that longer disease duration [27], UPDRS total score [27], working memory and verbal fluency [30] were also a predictors of this dimension. Finally, fatigue along with anxiety scores, were predictors of the bodily discomfort dimension. Other studies found that fatigue, UPDRS and female sex [27], anxiety [13, 17, 28], depression and hallucinations [17], and motor fluctuations [28] were predictors of this dimension. However, although in our study depression scores were included in the bodily discomfort model, anxiety and fatigue scores showed a higher association with this dimension, predominating over depression scores. In line with other studies [17, 28], social support dimension was predicted by anxiety scores and UPDRS-III. Additionally, anxiety scores also predicted the cognition dimension. PDQ-39 items of the cognition dimension assess concentration problems, the sensation of having a bad memory and hallucinations or nightmares, which generate feelings of anxiety or nervousness in PD patients. Even though we expected to find neurocognition as a predictor of this dimension, it did not happen. This may be due to the fact that the sensations of concentration problems and memory impairment approach a similar interpretation of cognition; while hallucinations or nightmares are more related to anxiety. In fact, one study suggests that the dimension of cognition in PDQ-39 has a stronger relationship with mood states rather than with neurocognitive domains [63]. Interestingly, neurocognition was found as a predictor of communication dimension, in addition to motor symptoms. The range of communication impairment is very variable in PD (from a lack of problems to inaudible and unintelligible speech) [64]. Indeed, acoustic speech deficits, use of action verbs and pausing have been shown to be more associated with motor impairment and linguistic deficits with cognitive impairment [64].

Concerning stigma, no significant associations were found, so they could not be included in the model. In contrast, other studies showed that anxiety [13, 28], reduced activity and motivation [27], and depression [31] predicted the stigma dimension in PD. Low scores on the stigma dimension may explain the lack of association with the rest of the variables. Furthermore, as Tu and colleagues noted [31], understanding stigma requires a broader consideration from a more social context, and not only focusing on motor and non-motor symptoms. Regarding the rest of the variables, our results did not show a significant association between apathy scores and HRQoL or any of its dimensions. However, other studies revealed an association of apathy with poorer HRQoL [17, 60] and with an increased self-rating of cognition and communication difficulties [17].

Recognition and treatment of motor and non-motor symptoms in the early stages of the disease may improve the HRQoL of PD patients. Considering that PD is a physically and psychologically debilitating disorder, health care professionals should adopt a holistic approach to PD rehabilitation [65]. Treatment objectives vary among individuals, highlighting the need for personalized intervention; so, a variety of non-pharmacological therapies should also be taken into account [66], such as, mindfulness yoga [65], psychological interventions [67], spiritual resilience [68], physical therapy [69], cognitive training [70], and speech therapies [71]. These therapies could be included in the clinical manage of PD patients.

It should be noted that the results of our study were based on cross-sectional data, limiting the ability to observe the impact of motor and non-motor symptoms on HRQoL in the disease course. For future research, longitudinal analyses would be needed to determine if the predictive symptom intervention could affect HRQoL. In fact, a recent longitudinal study examining predictors of HRQoL impairment in PD patients showed that, after a 2-year follow-up, age, sex, mood, and non-motor impairment were associated with clinically significant HRQoL impairment in PD patients [72]. On the other hand, the HRQoL evaluated using PDQ-39 does not allow to contrast the results with other pathologies, since the PDQ-39 is a specific PD scale. In this study, PD patients did not have a long disease duration and were mildly to moderately affected, without major impairments in their HRQoL, so it could be interesting to analyze the predictors of HRQoL and its dimensions in more advanced stages of the disease. Additionally, the low sample size limits generalization, so it would be interesting to conduct future studies with larger sample sizes. In fact, our study was carried out in a Spanish population and there are studies using PDQ-8, which is an abbreviated scale of PDQ-39, suggesting that geographic location has an effect on the non-motor symptoms affecting HRQoL [73]. Specifically, mood and sleep were the major predictors in European patients, whereas, non-motor symptoms were not significant predictors of HRQoL in Indian and Japanese patients [73] and in Chinese population anxiety, depression, motor symptoms, and marital status were the main predictors of HRQoL [74].

In conclusion, our results showed that anxiety, fatigue, motor symptoms, and depression were the main predictors of HRQoL total index in PD patients, whereas, after removing emotional well-being overlapping items, fatigue, anxiety, motor symptoms, and neurocognition were the predictors of HRQoL total index. These findings indicate the importance of identifying and controlling the overlap of items between HRQoL measures and clinical variables in order to perform an accurate interpretation of the results. Our results and methodology would be extrapolated to other pathologies. Additionally, results showed the impact of fatigue, anxiety, motor symptoms, and neurocognition scores in HRQoL dimensions in PD patients. Consequently, results suggest the importance of the appropriate assessment of HRQoL and its specific dimensions, because predictors are different for each dimension of HRQoL. Recognition and management of motor and non-motor symptoms in the early stages of the disease are essential, as these features have a greater impact on the HRQoL of people with PD. Therefore, intervention on fatigue, anxiety, motor symptoms, and cognitive processes may be a crucial target to improving HRQoL in PD patients.

## Supplementary Information

Below is the link to the electronic supplementary material.

Supplementary file1 (DOCX 81 kb)

## Data Availability

The data that support the findings of this study are available on reasonable request from the corresponding author.
