# Supplementary material for: Predictors of health-related quality of life in Parkinson’s disease: the impact of overlap between health-related quality of life and clinical measures
Source: Qual Life Res. 2022 Jul 16;31(11):3241–52. doi: 10.1007/s11136-022-03187-y (PMC9546987; doi:10.1007/s11136-022-03187-y)
Supplement: Supplementary file 1 — Supplementary file1 (DOCX 81 kb) [file 11136_2022_3187_MOESM1_ESM.docx]

**Supplementary material**

**Figure 1:** Spearman's rho significant correlation analyses of the HRQoL total index

**
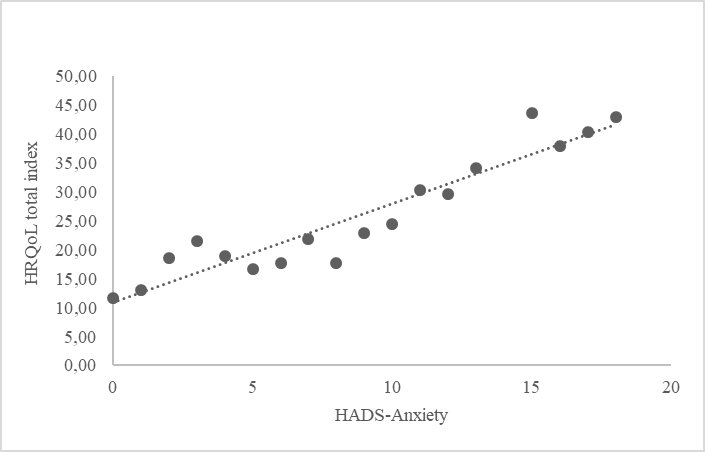
**

**
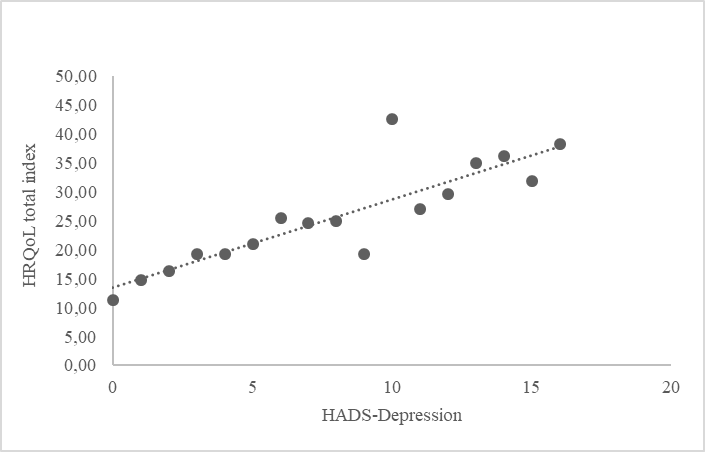
**


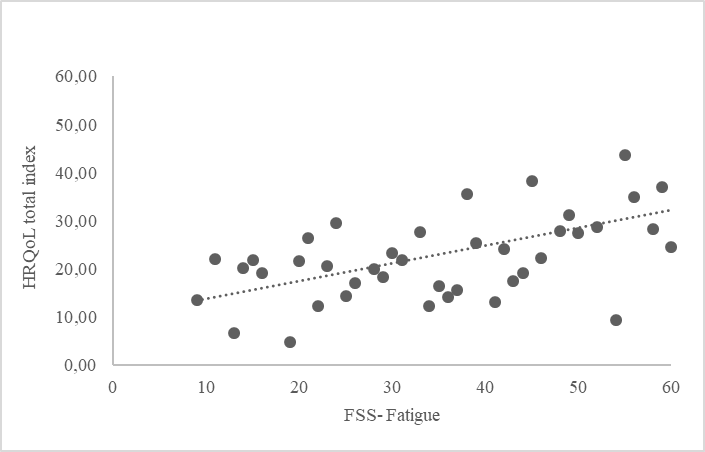


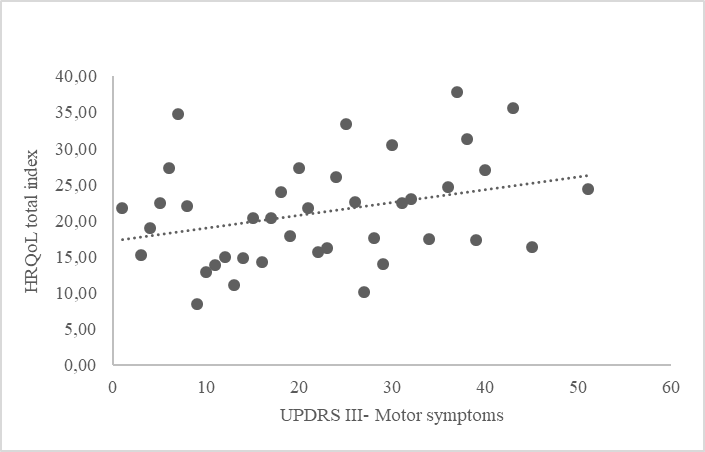


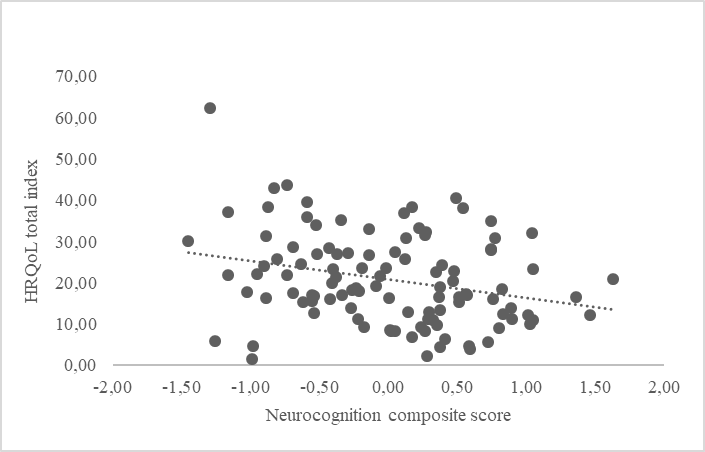


Legend: HRQoL= Health-Related Quality of Life; HADS= Hospital Anxiety and Depression Scale- Anxiety and Depression scores; FSS= Fatigue Severity Scale; UPDRS III= Unified Parkinson's Disease Rating Scale- motor part.
